# Single- and double-stranded DNA binding proteins act in concert to conserve a telomeric DNA core sequence

**DOI:** 10.1186/2041-9414-2-2

**Published:** 2011-01-14

**Authors:** Jenny Rhodin Edsö, Cecilia Gustafsson, Marita Cohn

**Affiliations:** 1 Department of Biology, Lund University, Biology building, Sölvegatan 35, SE-223 62 Lund, Sweden

## Abstract

**Background:**

Telomeres are protective cap structures at the ends of the linear eukaryotic chromosomes, which provide stability to the genome by shielding from degradation and chromosome fusions. The cap consists of telomere-specific proteins binding to the respective single- and double-stranded parts of the telomeric sequence. In addition to the nucleation of the chromatin structure the telomere-binding proteins are involved in the regulation of the telomere length. However, the telomeric sequences are highly diverged among yeast species. During the evolution this high rate of divergency presents a challenge for the sequence recognition of the telomere-binding proteins.

**Results:**

We found that the *Saccharomyces castellii *protein Rap1, a negative regulator of telomere length, binds a 12-mer minimal binding site (MBS) within the double-stranded telomeric DNA. The sequence specificity is dependent on the interaction with two 5 nucleotide motifs, having a 6 nucleotide centre-to-centre spacing. The isolated DNA-binding domain binds the same MBS and retains the same motif binding characteristics as the full-length Rap1 protein. However, it shows some deviations in the degree of sequence-specific dependence in some nucleotide positions. Intriguingly, the positions of most importance for the sequence-specific binding of the full-length Rap1 protein coincide with 3 of the 4 nucleotides utilized by the 3' overhang binding protein Cdc13. These nucleotides are very well conserved within the otherwise highly divergent telomeric sequences of yeasts.

**Conclusions:**

Rap1 and Cdc13 are two very distinct types of DNA-binding proteins with highly separate functions. They interact with the double-stranded vs. the single-stranded telomeric DNA via significantly different types of DNA-binding domain structures. However, we show that they are dependent on coinciding nucleotide positions for their sequence-specific binding to telomeric sequences. Thus, we conclude that during the molecular evolution they act together to preserve a core sequence of the telomeric DNA.

## Background

The ends of eukaryotic chromosomes form specialized chromatin structures called telomeres, which protect the chromosome ends from being degraded or recognized as double-strand breaks by the DNA damage response pathway. The assembly of the protective telomere cap structure is nucleated by the sequence-specific proteins binding to the double-stranded telomeric DNA and the single-stranded 3'-overhang, respectively. In addition to the shielding and protective role, the telomere binding proteins take part in a molecular mechanism which regulates the telomere to a species-specific length. In budding yeast, telomere length homeostasis is regulated by several proteins recruited to the telomere by the specific binding of Rap1 to the double-stranded telomeric DNA [1]. Rap1 binds DNA as a monomer via its two Myb-like homeodomain motifs present in the internally placed DNA-binding domain [1-4]. The functional analogs Trf1 and Trf2 in humans, and Taz1 in *Schizosaccharomyces pombe *contain a single Myb-like motif and hence can only bind DNA as dimers [5,6].

The species-specific telomeric DNA sequences are produced by the enzyme telomerase, which adds TG-rich repeats to the single-stranded 3' overhang by copying a template region in the telomerase RNA moiety [7]. The telomerase extension is regulated by the telomere binding proteins, which are involved in either the recruitment of telomerase or the blocking of telomerase access to the 3'-end. Rap1 regulates telomere length by mediating a protein counting mechanism via its interaction to Rif1 and Rif2 [8,9]. It has been shown that the length of the telomere is determined by the number of Rap1 binding sites [8,9]. When the telomere is short, few Rap1 proteins will bind and it is thought that the telomere will be in a telomerase accessible state. Conversely, when the telomeric DNA is extended and more Rap1 proteins can bind, the telomere is suggested to fold into a higher-order complex which is non-accessible for telomerase [10]. Still, however, the molecular details for this regulatory mechanism are not fully unravelled.

The single-stranded telomeric 3' overhang is protected by the highly sequence-specific protein Cdc13 in budding yeast [11-13]. Functional analogs in other species include TEBP in ciliates and Pot1 in vertebrates, plants and fission yeast [4,14]. Despite the divergence in sequence, they all bind the telomeric DNA via an OB-fold (oligonucleotide/oligosaccharide binding) domain [4,14]. Cdc13 positively regulates telomere length by recruiting the telomerase enzyme to the 3'end via its interaction with the telomerase subunit Est1 [15]. However, Cdc13 also associates with Stn1 and Ten1 to form the trimeric CST complex, a telomere-specific RPA-like complex which functions to negatively regulate telomere length [16].

The molecular evolution of the gene encoding the telomerase RNA has been shown to be extremely rapid [17-19]. While most other genes are clearly related between yeasts of the Saccharomyces, Candida and Kluyveromyces genera even on the nucleotide sequence level, the telomerase RNA genes have changed beyond sequence recognition and need to be identified via bioinformatic tools analyzing for a conserved secondary structure. This in turn provides a potential for a very high diversity of the telomeric sequences, which manifests among the budding yeast species where the telomeric repeats show an astonishing variation, ranging from 8 nucleotides in *Saccharomyces castellii *(syn. *Naumovia castellii*) to 26 nucleotides in *Saccharomyces kluyveri *(syn. *Lachancea kluyveri*) [20]. However, despite the remarkable sequence divergence, we have shown that Rap1 is able to bind the double-stranded telomeric DNA of these very distantly related yeast species [21,22]. In order to elucidate the prerequisites for this sequence-specific binding of Rap1 to telomeric DNA, we aimed for the determination of the minimal binding site (MBS) and the nucleotide positions of most importance for the specific binding of *S. castellii *Rap1 (scasRap1) to its cognate DNA target site. Furthermore, we wanted to elucidate whether the sequence specificity is retained within, and solely directed by, the DNA-binding domain of scasRap1. Determination of the exact binding site positions for the telomere proteins has implications for the functional analysis of how telomere chromatin assembly is established, and will enhance the analysis of the molecular mechanisms for the regulation of telomerase access to the 3'-end. Moreover, by comparing the binding sites and sequence specificities of Rap1 and Cdc13 in telomeric sequences of distantly related species, we are able to illuminate the molecular mechanisms shaping the evolution of telomeric DNA sequences.

## Results

### Rap1 binds a 12-mer minimal binding site in the telomeric DNA

**Figure 1:**
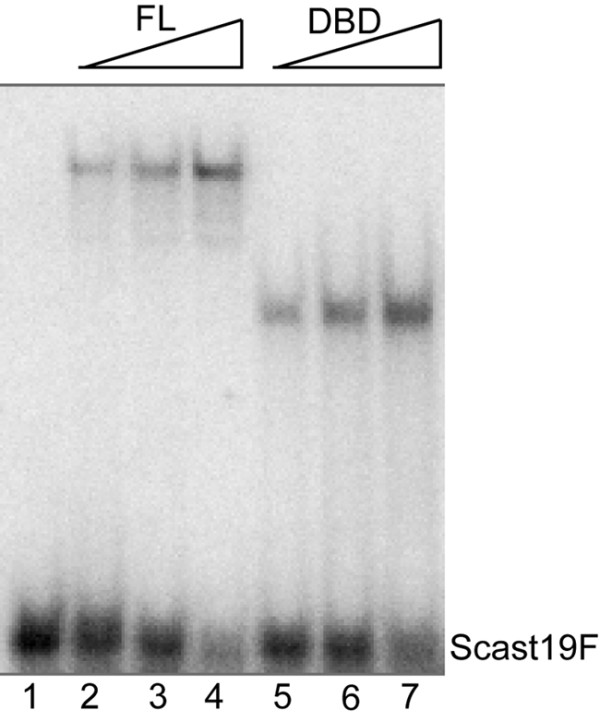
**The purified *Saccharomyces castellii *Rap1 DNA-binding domain (scasRap1-DBD) binds as efficiently to telomeric double-stranded DNA as the full-length protein**. EMSA of increasing amounts of the full-length scasRap1 (lanes 2-4) and scasRap1-DBD (lanes 5-7) incubated with the labeled double-stranded 19-mer telomeric DNA probe (Scast19F). No protein was added in lane 1. Protein was added in 2-fold increasing amounts.

The full-length *S. castellii *Rap1 (scasRap1) has previously been isolated [21]. To be able to analyze the inherent DNA binding properties of the DNA-binding domain (DBD), the DBD coding region of the scasRAP1 gene was isolated by PCR amplification of the DNA region corresponding to amino acids 336-582 (showing 78% identity to the *S. cerevisiae *RAP1 DBD) [2]. It was cloned, expressed in *E. coli *BL21 cells, and the purified scasRap1-DBD protein was run on SDS-PAGE to confirm the expected size (Additional file 1, Figure S1A). Using the Electrophoretic Mobility Shift Assay (EMSA), the scasRap1-DBD protein was found to bind efficiently to a 19-mer double-stranded telomeric oligonucleotide (Figure 1). Negative control extracts from bacteria containing empty pGEX-6p-1 vector did not show any shift in the EMSA (Additional file 1, Figure S1B). As expected, the complex produced by the much smaller scasRap1-DBD showed an increased mobility compared to the full-length scasRap1 protein. The binding was performed in a reaction containing high amounts of non-telomeric competitor DNA, and thus the purified *S. castellii *Rap1-DBD retains a similar high binding specificity for telomeric DNA as the full-length scasRap1 [21].

**Figure 2:**
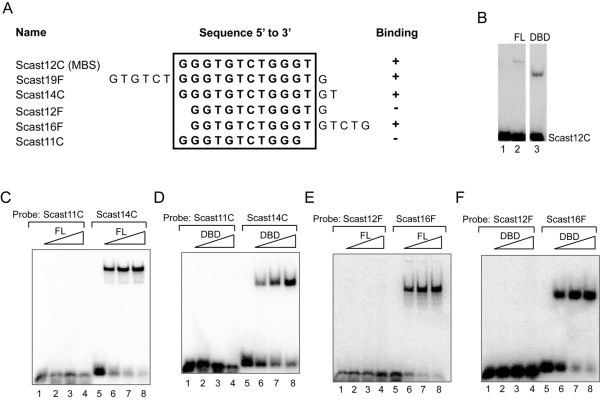
**The minimal binding site (MBS) of full-length scasRap1 and scasRap1-DBD is a 12-mer (Scast12C)**. (A) The ability of scasRap1 versus scasRap1-DBD to bind various oligonucleotides in the EMSA is indicated with + for binding and - for no binding. The MBS is enclosed by the box. (B) EMSA with the 12-mer MBS (Scast12C). Lane 1, no protein added; lane 2, full-length scasRap1; lane 3, scasRap1-DBD. (C-D) Both full-length scasRap1 (FL) and scasRap1-DBD (DBD) can bind to a double-stranded 14 nucleotide long telomere probe (Scast14C), but can not bind to an 11-mer lacking the last 3' nucleotide of the MBS (Scast11C). (E-F) The loss of binding to a 12-mer oligonucleotide lacking one nucleotide of the MBS in the 5'-end (Scast12F), is regained by an extensive five nucleotide elongation in the 3'-end (Scast16F). Protein was added in 2-fold increasing amounts. Lanes 1 and 5, no protein added.

To determine the minimal DNA binding site (MBS) of scasRap1, EMSA was performed with telomeric oligonucleotides of various length and permutation (Figure 2). The full-length scasRap1 and the scasRap1-DBD showed the same binding properties in these experiments and the MBS was determined as the 12-mer 5'- GGGTGTCTGGGT-3' for both proteins (Figure 2A-B). Shortening of the MBS by one nucleotide in either the 5' or the 3' end abolished the binding of both proteins (Figure 2). A compensatory lengthening of one nucleotide in the opposite end did not re-establish binding (Figure 2A, 2E, 2F; Scast12F, and data not shown). However, a one nucleotide shortening in the 5' end could be compensated by a substantial lengthening of five nucleotides in the 3' end (Figure 2A, 2E, 2F; Scast16F). This may be explained by the previously demonstrated ability of Rap1 to bind in a flexible way to the telomeric DNA [21-24]. The present results imply that this characteristic is inherent in the DNA-binding domain.

### Rap1 shows a sequence-specific DNA interaction with two 5 nt motifs

**Figure 3:**
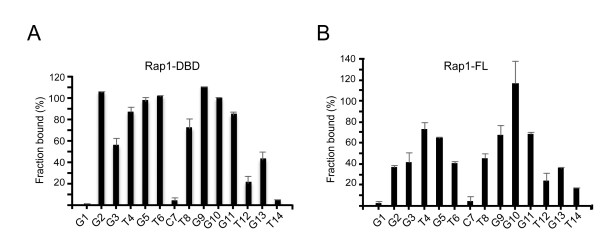
**The sequence specificity of Rap1 binding to telomeric DNA**. The impact of single-nucleotide substitutions on the binding ability of (A) scasRap1-DBD and (B) full-length scasRap1 was analyzed by EMSA competitions. The fraction of labeled wild-type probe still bound at an excess of non-labeled mutated competitor is depicted for each mutated position. A high value means that the position is important for the sequence-specific binding. Note that the absolute values should only be compared within the same graph. The wild-type oligonucleotide Scast14C (numbered sequence at the bottom of the graphs) was included in each gel and used to normalize the signal.

To determine which nucleotide positions are of importance for the sequence-specific DNA recognition of scasRap1, we analyzed the binding ability of scasRap1-DBD to telomeric sequences containing single-nucleotide mutations. All the 14 positions in the 14-mer Scast14C (Figure 2A) were subjected to transversions and used as cold competitors in EMSA reactions containing the labeled wild-type telomeric probe (Table 1, Additional file 1, Figure S2A). The summarized results show that the binding of scasRap1-DBD is not significantly impeded by mutations in positions G1, C7 or T14, while the interaction shows a medium to strong sequence dependency on the rest of the positions (Figure 3A). This pattern indicates the bi-partite structure of the protein, with the two Myb-like motifs each contacting one of the respective half-sites of the binding site [2,21]. The 5' half-site extends over 5 nucleotides (positions G2-T6), while the 3' half-site includes 6 nucleotides (positions T8-G13). Notably, this is resembling the situation for *S. cerevisiae *Rap1-DBD, where two motifs of 5 versus 6 nucleotides are bound by the domain 2 and domain 1, respectively [2,3]. Thus, for these two homologous telomeric proteins, the two subdomains of the DBD both use a more extended DNA interaction than the three nucleotides normally used by c-Myb and homeodomain protein-DNA complexes.

**Table 1:** Oligonucleotides used in this study

Name	Sequence 5' to 3'	Length (nt)
Scast10C	GGGTGTCTGG	10
Scast11C	GGGTGTCTGGG	11
Scast12C	GGGTGTCTGGGT	12
Scast12F	GGTGTCTGGGTG	12
Scast14C	GGGTGTCTGGGTGT	14
Scast16F	GGTGTCTGGGTGTCTG	16
Scast19F	GTGTCTGGGTGTCTGGGTG	19
Scast14C1	**C**GGTGTCTGGGTGT	14
Scast14C2	G**C**GTGTCTGGGTGT	14
Scast14C3	GG**C**TCTCTGGGTGT	14
Scast14C4	GGG**A**GTCTGGGTCT	14
Scast14C5	GGGT**C**TCTGGGTGT	14
Scast14C6	GGGTG**A**CTGGGTGT	14
Scast14C7	GGGTCT**G**TGGGTGT	14
Scast14C8	GGGTGTC**A**GGGTCT	14
Scast14C9	GGGTGTCT**C**GGTGT	14
Scast14C10	GGGTGTCTG**C**GTGT	14
Scast14C11	GGGTCTCTGG**C**TGT	14
Scast14C12	GGGTGTCTGGG**A**CT	14
Scast14C13	GGGTGTCTGGGT**C**T	14
Scast14C14	GGGTGTCTGGGTG**A**	14

Scast12C represents the minimal binding site (MBS). Bold letters in Scast14C1-Scast14C14 indicate mutated positions.

When we performed the same mutation analysis of the full-length scasRap1 protein, we found a similar overall pattern with the two half-sites split by the C7 position (Figure 3B and Additional file 1, Figure S2B). Thus, we could conclude that the isolated DBD has retained the same binding site characteristics as the full-length protein. Although both the full-length and DBD proteins show a marked sequence dependency on nine nucleotide positions of the MBS (G2-T6 and T8-G11), some differences in the details may be noted. For full-length scasRap1, mutations in positions T4-G5, and T8-G11 (underlined, 5'-GGGTGTCTGGGTGT-3') most severely affected the ability of the oligonucleotide to compete for binding (Figure 3B). Hence, these six nucleotides are of high importance for sequence-specific binding. However, for the scasRap1-DBD, all 9 nucleotides show a high importance for sequence specificity (underlined, 5'- GGGTGTCTGGGTGT-3') (Figure 3A). In view of this result, it is interesting to reflect on the presence of a linker region in between the two Myb-like motifs. This linker is unstructured when not bound to DNA, but is suggested to contribute to the binding of *S. cerevisiae *Rap1-DBD and to direct the C-terminal domain into its position [23,25]. However, there is currently no information on the structural status of this linker when present within the full-length *S. cerevisiae *Rap1. Possibly, the contribution of the linker may differ within the context of the full-length protein, thus giving rise to the minor variations seen between scasRap1 and scasRap1-DBD in our mutation analyses.

In summary, we found that the isolated scasRap1-DBD possesses similar binding properties as the full-length scasRap1 to double-stranded telomeric DNA. They share the same 12-mer MBS, and bind with the same spacing and similar sequence dependency to two half-sites of the binding site. These data show that the sequence specificity of Rap1 to the double-stranded telomeric DNA is fully directed by the two internally located Myb-like motifs comprising the DNA-binding domain.

### The Rap1 and Cdc13 sequence specificities coincide and are highly conserved in evolution

**Figure 4:**
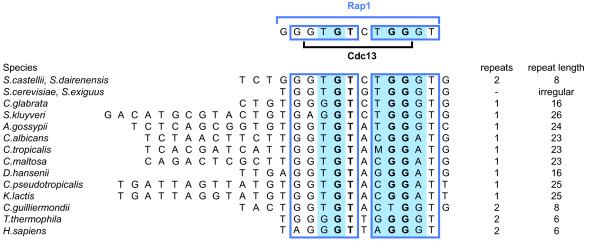
**The Rap1 binding site is highly conserved through telomere sequence evolution and overlaps with the Cdc13 binding site sequence**. Upper part, the 12-mer MBS of scasRap1 is indicated with the upper blue bar, and boxes showing the two 5 nt half-sites. Highlighted blue areas indicate the six nucleotides most important for sequence-specific binding of scasRap1. Lower black bar indicates the 8-mer MBS of scasCdc13 and bold letters indicate the four nucleotides most important for sequence-specific binding of scasCdc13. Three of these positions coincide with those most important for scasRap1 sequence specificity (bold letters in blue area). Lower part, alignment of the highly divergent telomeric sequences of different yeast species. The three nucleotides where the sequence specificity of Rap1 and Cdc13 coincide (blue+bold) are highly conserved. M indicates A or C. The genera included are Saccharomyces (S.), Candida (C.), Ashbya (A.), Debaryomyces (D.) and Kluyveromyces (K.). *Homo sapiens *and *Tetrahymena thermophila *are included since both scasRap1 and scasCdc13 bind to these sequences. Many fungi have the same telomere sequence as *H. sapiens *[37].

The Cdc13 proteins function to protect and regulate the length of telomeres in budding yeast [11-13,16]. Cdc13 binds to single-stranded telomeric 3' overhangs with high sequence specificity via a DBD containing an oligonucleotide/oligosaccharide-binding domain (OB-fold) [11,26]. Thus, in spite of their binding to different parts of the telomere, Cdc13 and Rap1 both depend on the binding to the same DNA sequence in order to fulfill their cellular functions. This prompted us to compare their respective DNA sequence specificities. We previously determined the MBS of *S. castellii *Cdc13 (scasCdc13) as the 8-mer 5'-GTGTCTGG-3', with four nucleotides being the most important for sequence-specific binding (5'-GTGTCTGG-3') (Figure 4) [12,13]. Intriguingly, when superimposing the respective binding sites of the two proteins, we found that the MBS of scasCdc13 is completely and symmetrically enclosed within the 12-mer MBS of scasRap1 (Figure 4, upper schematic).

We have shown that *S. cerevisiae *Rap1, *S. castellii *Rap1 and *S. dairenensis *Rap1 bind telomeric sequences from various yeast species [21,22,24]. Similarly, scasCdc13 binds the telomeric sequences of distantly related yeast species [12,13]. When aligning the telomeric repeats of various yeast species, we find that a core motif corresponding to the Rap1 and Cdc13 binding sites is highly conserved within the otherwise divergent sequences (Figure 4, lower part). The two half-sites of the Rap1 binding site (boxes) are easily distuinguished with all the five positions of the 5' half-site and three positions of the 3' half-site being highly conserved through the telomeric sequences of most yeast species. The four nucleotides which are most important for Cdc13 binding constitute a GT-GG motif, where the GT dinucleotide is located inside the 5' half-site and the GG dinucleotide is located inside the 3' half-site of the Rap1 binding site (Figure 4, bold letters). Of the six positions that are most important for the sequence specificity of full-length Rap1 (highlighted blue area), three of them coincide with the Cdc13 GT-GG motif. Intriguingly, those three overlapping positions show a higher degree of conservation than the three that do not coincide, implying that they have been kept under pronounced selection pressure.

## Discussion

The sequence-specific telomere binding proteins are essential for the establishment of a protective telomere chromatin structure. They are also taking part in the regulation of the telomerase elongation of the 3'overhang. In this report we show that the sequence-specific telomeric DNA recognition by the full-length scasRap1 depends on the interaction with two 5 nt half-sites, having a 6 nt centre-to-centre spacing. The scasRap1 DNA-binding domain (DBD) is established by two Myb-like homeodomain helix-turn-helix structures connected by an extended linker region. We show that the sequence-specific DNA binding properties of scasRap1 are completely inherent in the isolated DBD. It is therefore very likely that each half-site is contacted by one of the Myb-like motifs of the DBD. Furthermore, we have previously shown that the sequence specificity of scasCdc13 is completely retained within its isolated DBD. The Cdc13 DBD consists of an OB-fold domain which forms a closed β-barrel structure by two orthogonally packed antiparallel β sheets [2,26]. Thus, the Rap1 and Cdc13 proteins encompass two very distinct types of DBD structures. Nevertheless, we find they specifically recognize the very same core sequence within the double- vs. single-stranded telomeric DNA. Moreover, it is highly intriguing that the two half-sites of the two respective DNA binding sites coincide.

**Figure 5:**
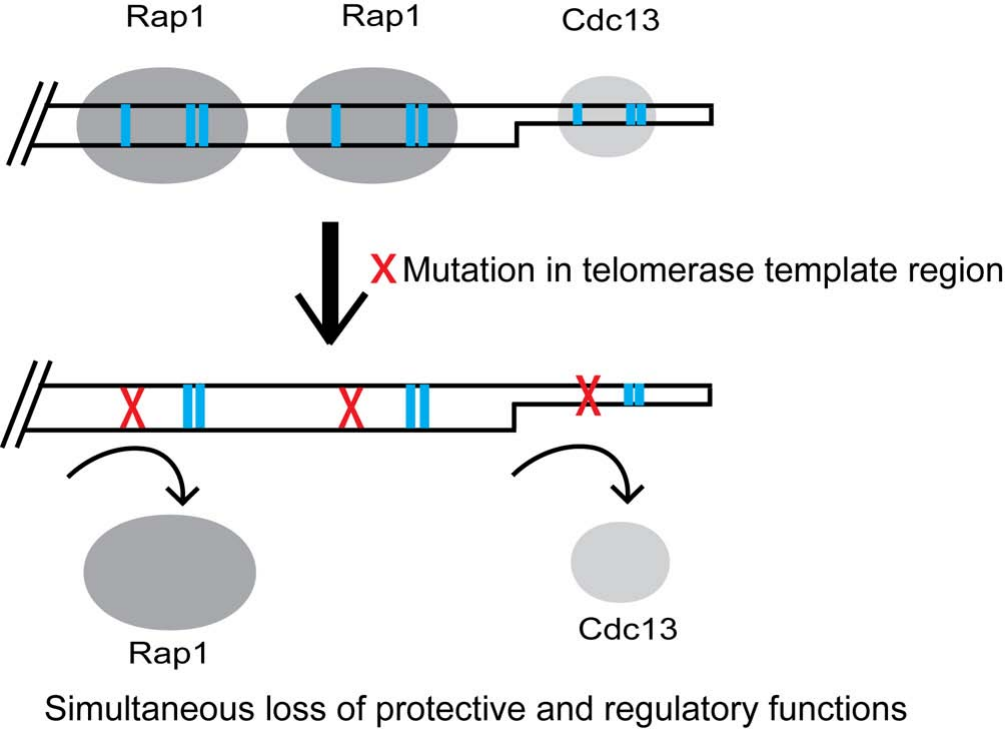
**Simultaneous loss of Rap1 and Cdc13 binding would lead to drastic dysregulation of telomeres**. Rap1 binds to the double-stranded part and Cdc13 binds to the single-stranded part of the telomere. In this paper we show that their binding site sequences coincide. The blue bars indicate the three nucleotides that are shared between Rap1 and Cdc13 among those positions of highest importance for their respective binding. If one of these three positions is mutated it would lead to a simultaneous loss of binding for both Rap1 and Cdc13, which would be predicted to have a severe effect on the telomere protection and length regulation.

The telomerase RNA components from different organisms range in size considerably, and the sequence is highly diverged [17-19]. This remarkably high rate of molecular evolution of the telomerase RNA genes provides the potential for a similarly high rate of change in the telomeric repeats. This is evident by the highly divergent telomere sequences of the budding yeasts, having a range of 8-26 bp repeats (Figure 4). However, we have here unveiled a molecular mechanism where two distinct types of proteins are involved in the evolutionary conservation of a single core motif within the telomeric repeats. Although the telomeric repeats are highly diverged, the three nucleotides where the sequence specificity of Cdc13 and Rap1 coincide are highly conserved, thus ensuring the proper binding and functionality of both proteins. A mutation in one of these positions would lead to a simultaneous impairment of the Rap1 and Cdc13 interactions with DNA, and consequently would severely affect the telomere function (Figure 5).

With the here presented data on the sequence specificity of Rap1 in compilation with the Cdc13 data, it will now be possible to make a more detailed examination of the respective contributions of the Rap1 and Cdc13 proteins in the telomere length regulation mechanism. For example, our results shed light upon previous observations where a drastic telomere lengthening phenotype was seen for *Kluyveromyces lactis *cells when mutations were introduced into the telomeric repeats [27]. This loss of telomere length control was attributed to the loss of Rap1 binding. A base change in the position T4 of the Rap1 MBS (Figure 4, upper part), which is highly important for Rap1 but not for Cdc13, caused a gradual telomere lengthening over several passages on plates [27]. This finally led to abnormally long telomeres and reduced growth. However, a mutation in the G5 position (Figure 4, upper part) caused a much more extreme, immediate phenotype, with abnormally long telomeres appearing already in the the first passage [27]. Since both the Rap1 and Cdc13 proteins are dependent on the G5 position for their sequence-specific binding, this implies that loss of Cdc13 binding contributes to the immediate dramatic telomere lengthening phenotype. Possibly, this may be explained by the loss of Cdc13 proteins leaving the 3'overhang unprotected and available for the extension by telomerase. Furthermore, our results provide a basis for understanding the gradual lengthening of telomeres that was observed when human telomeric sequences were added onto *S. cerevisiae *telomeres by expressing the human telomerase RNA gene [28]. These so-called humanized telomeres undergo a progressive replacement of the *S. cerevisiae *TG_1-3 _sequences by the human T_2_AG_3 _repeats. In the alignment of the human telomeric sequence to the yeast sequences, we note that the first nucleotide in the respective highlighted Rap1 motifs are differing from the *S. cerevisiae/S. castellii *sequence (Figure 4, positions T4 and T8 in Rap1 MBS). While changes in both of these two positions would be predicted to greatly reduce the Rap1 binding, they should not affect the Cdc13 binding. In accordance with this, *S. cerevisiae *Rap1 shows a very weak binding to the human T_2_AG_3 _repeats [29], while Cdc13 shows a rather high binding activity to T_2_AG_3 _repeats [30]. The situation in the humanized telomeres therefore corresponds to the *K. lactis *strain mutated in position T4 of the Rap1 MBS. Thus, we can conclude that *S. cerevisiae *and *K. lactis *show a similar *in vivo *effect, with gradual telomere lengthening, when nucleotide positions important for Rap1 sequence-specific binding are mutated.

The heterogeneous telomeric sequences of *S. cerevisiae *constitute a major difficulty when aiming for the estimation of the total number of Rap1 binding sites along a telomeric array. Presumably, each telomere contains a different distribution of high and low affinity binding sites. It was recently shown that Rap1 binds independently to each binding site in a non-cooperative manner, implying that Rap1 monomers load onto and release each site within an array without regard to the occupancy of the other sites [31]. Moreover, it was shown that the Rap1 *in vitro *binding affinity correlates with the ability of telomeric repeat arrays to regulate telomere length *in vivo*. Our data presented here will provide a better opportunity to pinpoint the high affinity binding sites within a given *S. cerevisiae *telomere array, which will be helpful in the determination of the correlation between the number of Rap1 binding sites and the telomere length.

The origin and molecular evolution of telomeres still remains obscure. It has been suggested that linear chromosomes were created by transposable elements that invaded an ancient circular chromosome and thereby provided a mechanism for maintenance of linear chromosomes [32]. In fact the telomeres of *Drosophila *are maintained by very long non-LTR retrotransposons that evolve faster than euchromatic genes [33]. Whether they would represent a more ancient type of telomere repeats, and whether the evolution of telomeric repeats has drifted in a unison direction, are very interesting questions remaining to be answered. In any way, in an evolutionary perspective the mechanisms for the maintenance of the chromosome ends are apparently very dynamic and flexible cellular processes. However, the loss of binding of any functionally important protein will be highly efficient in providing a conservational force acting against the genetic drift. Here we see that a single point mutation may simultaneously affect the binding of two of the telomere-binding proteins having major functions in the protection and length regulation of yeast telomeres (Figure 5). Our finding has implications for the future research on telomere maintenance, because it will allow the separation of function of Rap1 vs. Cdc13 in telomere protection and length regulation and will enable a better interpretation of the result.

## Conclusions

We found that *S. castellii *Rap1 binds a 12-mer minimal binding site within the double-stranded telomeric DNA and that the sequence specificity is dependent on the interaction with two 5 nt motifs. The nucleotide positions of most importance for the sequence-specific binding coincide with three of the four nucleotides important for the specificity of the 3' overhang binding protein Cdc13. These nucleotides are very well conserved within the otherwise highly divergent telomeric sequences of yeasts. Thus, although containing very distinct types of DNA-binding domain structures, the telomere-binding proteins Rap1 and Cdc13 act together to preserve a core sequence of the telomeric DNA.

## Methods

### Species designations and telomere sequences

*S. castellii *syn. *Naumovia castellii *and *S. dairenensis *syn. *Naumovia dairenensis *[34]. See Kurtzman 2003 [34] for new designations of the other genera in Figure 4. References for telomere sequences are found in [35,36] and as listed in Cohn et al. 2006 [37].

### Cloning of S. castellii Rap1-DBD

The DBD of *S. castellii RAP1 *(*scasRAP1*) was identified by an alignment of the *S. cerevisiae*, *K. lactis *and *S. castellii *Rap1 amino acid sequences [21]. The cloned *scasRAP1-DBD *sequence corresponds to nucleotides 1008-1746 in the *scasRAP1 *gene (accession number in GenBank AF401990). This sequence was amplified from *S. castellii *Y320 genomic DNA using the primers Rap1-P1008-F:5'-CCAAACCCGGGTTTACCATCTCATAATAAAG-3' and Rap1-P1746-R:5'-GTTACTCTCGAGTCTCTGCCTCTTAATTGC-3'. The PCR product was purified and cleaved with *SmaI *and *XhoI *and then ligated into a *SmaI*-*XhoI *cleaved pGEX-6p-1 vector (GE Healthcare). The ligation product was transformed into *E. coli *DH5α cells and then correct clones were transformed into *E. coli *BL21 cells for expression. Cloning of full-length scasRap1 has been described earlier [21].

### Gene expression and protein purification

100 ml of LB medium with 200 μg/ml carbenicillin was inoculated with an over night culture of transformants and incubated at 30°C until OD_600 _= 0.5, then 1 mM IPTG was added and the culture was grown for another 4 hours at 25°C. After harvesting by centrifugation the pellet was stored at -20°C over night. The pellet was dissolved in 2 ml of lysis buffer (50 mM Tris-HCl pH 7.6, 1% (v/v) Triton-X-100, 2 mM EDTA, 10% (v/v) glycerol, 0.5 M NaCl, 5 mM DTT and 1x protease inhibitor cocktail (Roche)), lysozyme was added to a final concentration of 1 mg/ml and the sample was incubated on ice for 30 minutes. The cells were disrupted by sonication for 6x10 seconds and the lysate was cleared by centrifugation at 14000 rpm for 20 minutes. The crude protein extract was affinity purified using Glutathione Sepharose 4B (GE Healthcare) in batch according to the manufacturers protocol. The GST-fusion protein was bound to 200 μl GS4B at room temperature for 30 minutes, then 10 bed volumes of 1x PBS (140 mM NaCl, 2.7 mM KCl, 10.1 mM Na_2_HPO_4_, 1.8 mM KH_2_PO_4_, pH 7.3) was used to wash the slurry and 200 μl reduced Glutathione pH 8 (10 mM) to elute the protein from the matrix. A fraction of the fusion protein was cleaved with PreScission Protease (0.04 U/μl) (GE Healthcare) at 5°C over night. The protease cleaved scasRap1-DBD protein was 263 amino acids long (17 extra amino acids from the GST-tag and the use of a Stop codon in the pGEX-6p-1 vector) with a calculated molecular weight of 30.6 kDa. The purity and efficiency of cleavage was analyzed on Coomassie stained SDS-PAGE gels. Full length scasRap1 was expressed and purified as previously reported [21]. Negative control extracts were prepared in parallel from bacteria containing empty pGEX-6p-1 vector.

### Electrophoretic mobility shift assay

The binding characteristics of scasRap1-DBD and scasRap1 were analyzed by electrophoretic mobility shift assay (EMSA) with gel purified oligonucleotides. Various telomeric and non-telomeric probes were used in direct binding studies and in competition analyses. The sequence specificity was investigated by using mutagenized oligonucleotides as competitors added in 10x, 100x, and 1000x molar excess for scasRap1 and 20x, 200x and 2000x molar excess for scasRap1-DBD. The forward strand of the probe was radioactively labeled with [γ-^32^P] ATP using T4 polynucleotide kinase (AB Gene). The probes were annealed with a double molar excess of the complementary strand (1 mM Tris-HCl pH 8, 0.1 mM MgCl_2_) by boiling for 2 minutes and slowly cooled down. In the EMSA reactions 10 fmol probe, 1 μg non-specific poly (dI-dC) competitor or 1.5 μg of a mixture of *E. coli *gDNA, yeast t-RNA and salmon sperm DNA (0.5 μg each), binding buffer (final concentration 10 mM Tris-HCl pH 7.5, 7 mM MgCl_2_, 8% glycerol) and different amounts of protein were incubated for 15 minutes at 25°C. In the initial experiments purified extracts were compared to crude extract and were found to give the same DNA binding shift, while the negative control extract did not give any shift at all (Additional file 1, Figure S1B). Typically the purified proteins were used in a range of 0.6-1.2 μg full-length Rap1 and 0.15-0.7 μg Rap1-DBD, while the crude extracts were used in a range of 0.1-0.4 μg full-length Rap1 and 0.15-0.6 μg Rap1-DBD. For the mutation analyses purified extract was used for the full-length scasRap1 and crude extract was used for scasRap1-DBD. In competition experiments non-labeled oligonucleotides were added in increasing amounts prior to protein addition. The samples were loaded on a 4% (full-length) or 6% (DBD) non-denaturing polyacrylamide gel and run in 1xTBE (89 mM Tris-Borate, 2 mM EDTA pH 8.0), 150 V at 4°C. After drying the gel the signal was analyzed using a phosphor imager. The mutation analyses were performed as EMSA competitions where unlabeled mutated oligonucleotides were added and allowed to compete with the protein binding to the labeled wild-type probe. The fraction of bound probe without any competitor was set as 100% shift and the percent of probe still shifted at 1000 times molar excess (scasRap1) or 2000 times molar excess (scasRap1-DBD) of competitor was assessed. The shift was calculated as a ratio of the total signal per well (sum of the shifted and free probe) in order to normalize for any variation in loading volumes. The wild-type oligonucleotide was used to normalize the signal in each gel. Each experiment was repeated at least twice per modified oligonucleotide and the average result was calculated.

## Competing interests

The authors declare that they have no competing interests.

## Authors' contributions

JRE planned and supervised the experiments and wrote the paper. CG carried out the experiments and made the quantifications. MC initiated, planned and supervised the study and wrote the paper. All authors read and approved the final manuscript.

## Supplementary Material

Additional file 1 **Supplementary Figure 1 and Figure 2**. Two figures showing additional data. Click here for file
