# Supplementary material for: Single- and double-stranded DNA binding proteins act in concert to conserve a telomeric DNA core sequence
Source: Genome Integr. 2011 Jan 14;2:2. doi: 10.1186/2041-9414-2-2 (PMC3033795; doi:10.1186/2041-9414-2-2)
Supplement: Additional file 1 — Supplementary Figure 1 and Figure 2. Two figures showing additional data. [file 2041-9414-2-2-S1.PDF]

Supplementary material for:

Single- and double-stranded DNA binding proteins act in concert to conserve a telomeric DNA core sequence

Jenny Rhodin Edsö, Cecilia Gustafsson and Marita Cohn*

*To whom correspondence should be addressed. E-mail marita.cohn@cob.lu.se

Supplementary figures

A

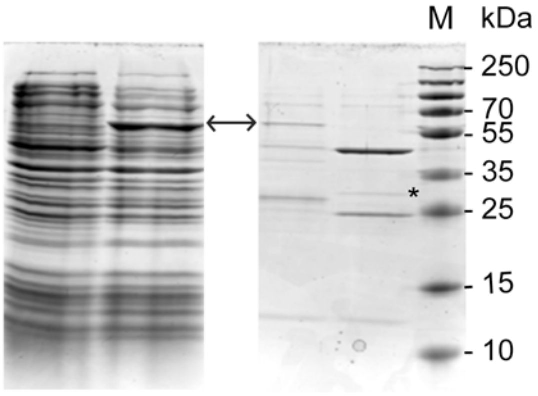

1 2 3 4 5

B

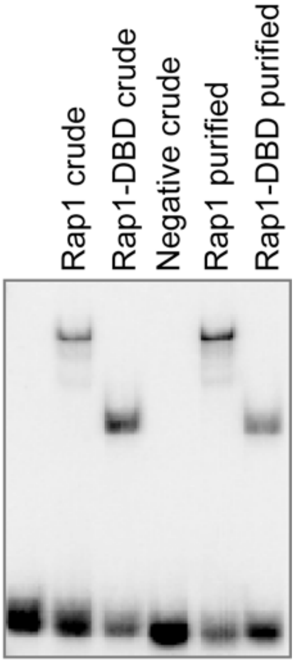

1 2 3 4 5 6

Figure 1. (A) Purification of the recombinant *Saccharomyces castellii* Rap1-DBD. ScasRAP1-DBD was expressed in *E. coli* and the extract was run on an SDS-PAGE to check for induction and cleavage of the GST-tag. Lane 1, crude extract before induction; lane 2, crude extract after induction; lane 3, purified extract; lane 4, purified extract where the GST-tag was completely cleaved off by PreScission protease. The arrows indicate the fusion protein (56 kDa) and the asterisk indicates cleaved protein (30 kDa). The PreScission protease (46 kDa) and the cleaved GST-tag (26 kDa) are clearly visible in lane 4.

(B) The crude extracts show the same specific binding as the respective purified scasRap1 and scasRap1-DBD proteins. The negative control extract with expression of empty pGEX-6p-1 vector does not show any shifted band. EMSA reactions with the labeled double-stranded telomeric DNA probe Scast19F. Lane 1, no protein added; lane 2, 0.3 μg of crude full-length scasRap1 extract; lane 3, 0.4 μg of crude scasRap1-DBD extract; lane 4, 1.5 μg of crude negative control extract; lane 5, 0.6 μg of purified full-length scasRap1; lane 6, 0.7 μg of purified scasRap1-DBD.

A

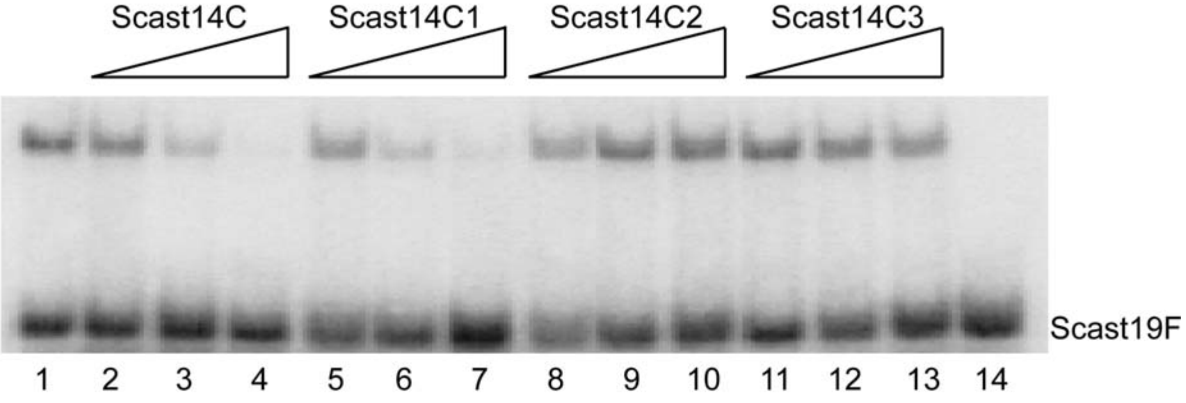

B

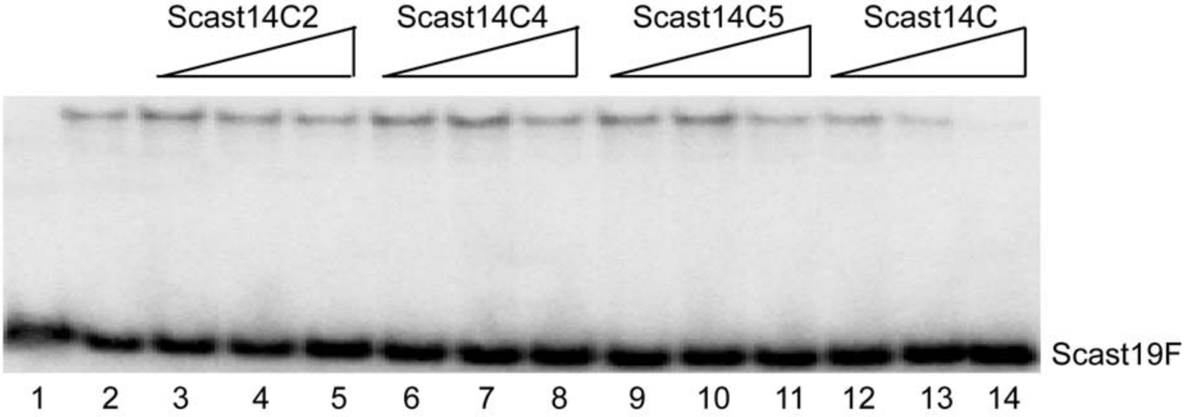

Figure 2. Analysis of the sequence-specific binding of scasRap1 and scasRap1-DBD by competing oligonucleotides with single nucleotide substitutions.

(A) ScasRap1-DBD was incubated with labeled Scast19F and the indicated non-labeled mutated competitors at 20, 200 and 2000 times molar excess. Lane 2, no competitor added; lane 14, no protein added. (B) Full-length scasRap1 was incubated with labeled Scast19F and mutated competitors at 10, 100 and 1000 times molar excess. Lane 1, no protein added; lane 2, no competitor added. Wild-type Scast14C was added as a control competitor in all gels and was used for the normalization of the quantifications.
